# QUALITY OF DAILY ACTIVITIES AND LONELINESS IN A ROMANTIC RELATIONSHIP

**DOI:** 10.1093/geroni/igad104.1417

**Published:** 2023-12-21

**Authors:** Jana Nikitin, Fabio Selovin, Fiona Rupprecht

**Affiliations:** University of Vienna, Vienna, Wien, Austria; University of Vienna, Vienna, Wien, Austria; University of Vienna, Vienna, Wien, Austria

## Abstract

Romantic relationships can be a source of both the most positive and the most negative experiences. Thus, partners can feel fully integrated or lonely in a romantic relationship, depending on the quality of the relationship. Cross-sectional and longitudinal studies have shown that loneliness (defined as a subjectively perceived deficiency in a person’s social relationship) is more pronounced in low-quality relationships. Little is known about daily sources of loneliness within a couple. To explore this question, we investigated whether low-quality activities in a partnership can lead to feelings of loneliness in daily situations. To examine this question, we analyzed data from 72 individuals (M age = 43 years, range = 18 - 82 years; 64% female) who provided 576 measurement occasions on seven consecutive days in which they were involved in activities with their romantic partners. Participants indicated how they experienced the activity in each situation (positively or negatively) and how lonely they felt. Results showed that participants who reported lower-quality activities with their partner reported higher levels of daily loneliness (47% explained variance); and on occasions when participants reported lower-quality activities than usual, they also reported higher levels of loneliness (25% explained variance). Moreover, individuals who reported higher levels of loneliness during the 7-day assessment benefited less from high-quality activities with their partner in terms of reduced loneliness. Thus, loneliness not only results from, but also contributes to, how people experience daily activities with their partners. Gender and age had no effect on these associations, suggesting generalizability of the results.

